# A 5-year longitudinal study of schistosomiasis transmission in Shian village, the Anning River Valley, Sichuan Province, the Peoples' Republic of China

**DOI:** 10.1186/1756-3305-4-43

**Published:** 2011-03-24

**Authors:** Rong Zhu, Darren J Gray, Aaron P Thrift, Gail M Williams, Yi Zhang, Dong-Chuan Qiu, Feng Zheng, Yue-Sheng Li, Jiagang Guo, Hong-Qing Zhu, Wei-Ping Wu, Robert S Li, Donald P McManus

**Affiliations:** 1 National Institute of Parasitic Disease, Chinese Centre for Disease Control and Prevention, Shanghai, PR China; 2 Department of Epidemiology, School of Public Health, Fudan University, Shanghai, PR China; 3 Griffith Health Institute, Griffith University, Australia; 4 MolecularParasitology Laboratory, Infectious Diseases Division, Queensland Institute of Medical Research, Herston, Brisbane, Queensland, Australia; 5 School of Population Health, University of Queensland, Australia; 6 Institute for Parasitic Diseases, Sichuan Centre for Disease Control and Prevention, Chengdu, PR China; 7 Hunan Institute of Parasitic Diseases, World Health Organization Collaborating Centre for Research and Control on Schistosomiasis in Lake Region, Yueyang, Hunan, People's Republic of China

## Abstract

**Background:**

*Schistosoma japonicum *is a major public health concern in the Peoples' Republic of China (PRC), with over one million people infected and another 50 million living in areas at risk of infection. Based on ecological, environmental, population genetic and molecular factors, schistosomiasis transmission in PRC can be categorised into four discrete ecosystems or transmission modes. It is predicted that the Three Gorges Dam (TGD) will impact upon the transmission of schistosomiasis in the PRC, with varying degree across the four transmission modes. We undertook longitudinal surveillance from 2002 to 2006 in sentinel villages both above and below the TGD across five provinces (Hunan, Jiangxi, Hubei, Anhui and Sichuan) to determine whether there was any impact of the TGD on schistosomiasis transmission during its construction. Here we present the results from a schistosomiasis-endemic village located above the dam in Sichuan Province.

**Results:**

Baseline results showed a human *S. japonicum *prevalence of 42.0% (95% CI: 36.6-47.5). At follow-up, results showed that the incidence of *S. japonicum *infection in the selected human cohort in Shian decreased by three quarters from 46% in 2003 to 11.3% in 2006. A significant (P < 0.01) downward trend was also evident in the yearly adjusted (for water contact) odds ratios. Over the four years of follow-up, the incidence of *S. japonicum *infection in bovines declined from 11.8% in the first year to zero in the final year of follow-up.

**Conclusions:**

The substantial decrease in human (75%) and bovine (100%) incidence observed in Shian village can probably be attributed to the annual human and bovine PZQ treatment of positives; as seen in drug (PZQ) intervention studies in other parts of PRC. If an increase in schistosome transmission had occurred as a result of the TGD, it would be of negligible size compared to the treatment induced decline seen here. It appears therefore that the construction of the TGD had virtually no impact on schistosomiasis transmission in Shian village over the period of study. Furthermore, contrary to previous reports from Sichuan downplaying the role of animals in human schistosome transmission, bovines may indeed play a role.

## Introduction

In the People's Republic of China (PRC), zoonotic schistosomiasis, caused by *Schistosoma japonicum*, is a chronic debilitating disease with 50 million people at risk of infection[1]. Endemic foci are located in the lake and marshland regions of Southern China (Jiangxi, Hunan, Jiangsu, Anhui and Hubei provinces), where the majority of transmission occurs, and in the hilly and mountainous regions of Sichuan and Yunnan[1]. Based on ecological, environmental, population genetic and molecular factors, Davis *et al. *[2] categorised schistosomiasis transmission in the PRC into four discrete ecosystems or transmission modes. These are represented by Poyang Lake (mode I), Dongting Lake (mode II), the Yangtze River isles of Anhui (mode II), the canals and water networks of Hubei (mode III), and the hilly and mountainous areas of Sichuan and Yunnan (mode IV)[2].

**Figure 1:**
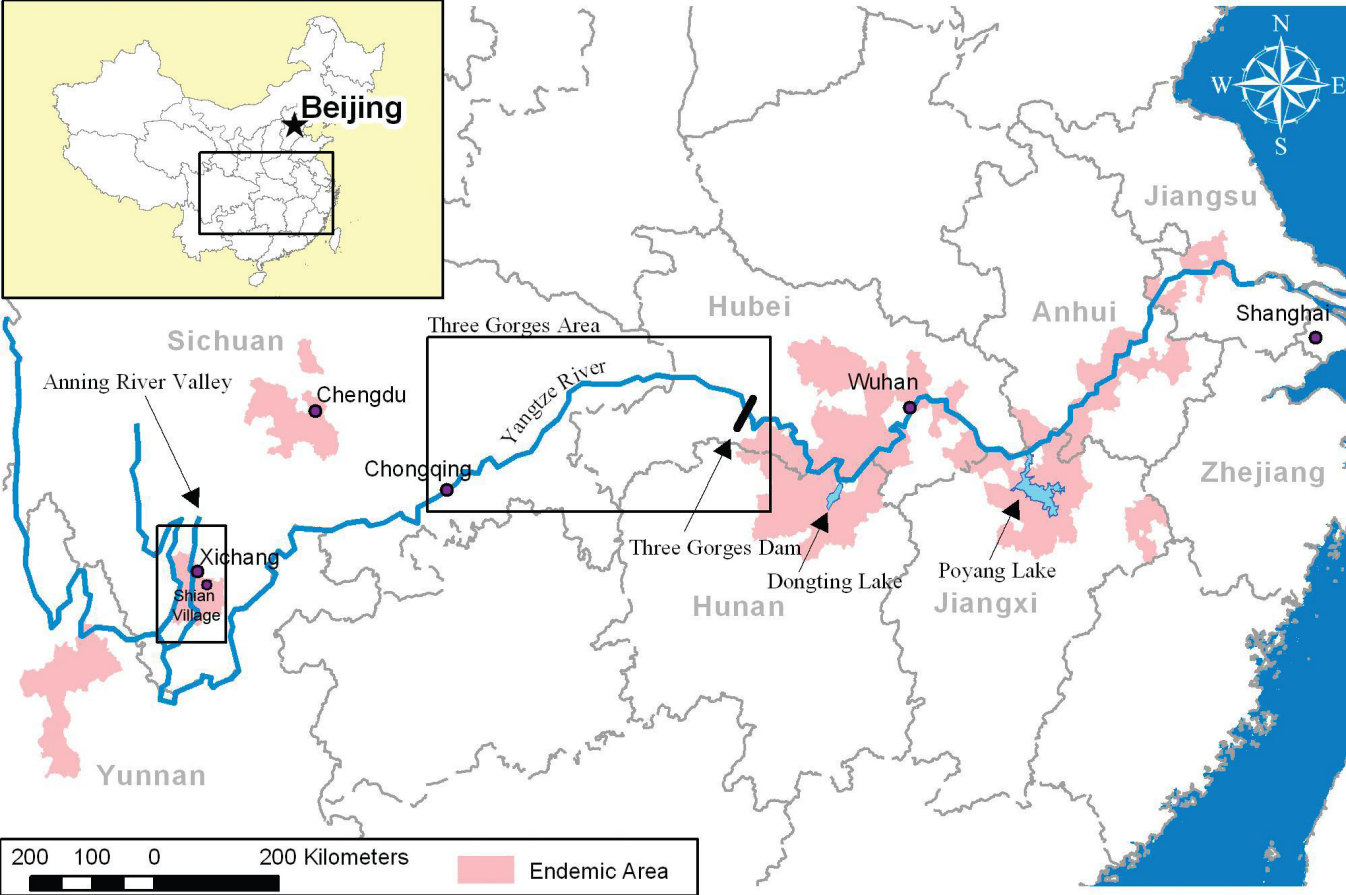
**Map of schistosomiasis endemic regions and the Three Gorges Dam, China showing the Anning River Valley and Shian village**.

It is predicted that the Three Gorges Dam (TGD) will impact upon the transmission of schistosomiasis in the PRC, with varying degree across the four transmission modes[3,4]. Transmission Modes I-III are located below the dam and mode IV is above the dam. The dam is located in the Three Gorges region in the upper reaches of the Yangtze River (the world's third-largest river; 5920 km long) (Figure 1). It spans the Yangtze at Sandouping Island, just west of the city of Yichang in Hubei province[1,3,4]. It is the world's largest hydroelectric project aimed at developing and controlling the Yangtze River, reducing flooding in the lower plains regions, and hence ameliorating economic losses[1,3,4]. Construction commenced in 1994 and by 2003 the TGD was closed to a height of 135 metres. In 2009, it reached its full height of 185 metres and began to generate 18,600MW of power for the whole of the PRC[5]. The dam will also help to control the lower Yangtze which is prone to periodic flooding. By 2009, the 2,300 m long dam resulted in a 600 km long reservoir that inundated 115,000 acres of cultivated land, requiring resettlement of some two million people[5].

We undertook longitudinal surveillance from 2002 to 2006 in sentinel villages both above and below the TGD across five provinces (Hunan, Jiangxi, Hubei, Anhui and Sichuan) to determine whether there was any impact of the TGD on schistosomiasis transmission during its construction. Here we present the results from a schistosomiasis-endemic village located above the dam in Sichuan Province.

## Methods

### Study design

We undertook a prospective longitudinal cohort study in the residents of Shian village, located at 27.84284N, 102.39277E, in the Anning River Valley, Sichuan Province, PRC (Figure 1) between 2002 and 2006, to determine the impact, if any, of the construction of the TGD on schistosome transmission. The primary end point measured was human incidence.

### Baseline

At baseline, two stool samples were collected and a questionnaire administered to all residents (N = 319) who usually resided in the village. Stool samples were examined microscopically using the Kato-Katz thick smear technique, with three slides per stool read blind, to determine *S. japonicum *prevalence and intensity of infection[6]. The questionnaire consisted of questions relating to demographics, medical history and water contact.

A stool sample was also collected from all bovines in Shian village and examined for *S. japonicum *prevalence using the miracidial hatching test (3 individual hatches read blind - 50 grams faeces/hatching) and intensity of infection, using a traditional Chinese sedimentation method[7].

### Follow-up and study procedures

Following the baseline survey, a fixed cohort of villagers (N = 285) was selected and monitored for schistosome infections for the duration of the study. The cohort inclusion criteria were that an individual must be: a) a resident of the village for more than 12 months; b) aged 5-65 years; d) not intending to migrate out of the village for the next 4 years; e) continuously reside in the study area over the study period. With a final cohort size of 240 residents and a 95% confidence interval for infection rates we will have a half-width of 3.15%.

A water contact questionnaire was administered to the cohort members annually; and consisted of general questions relating to participant yearly water exposure by season; as well, a month-long water contact diary was kept by each individual[8].

Two stool samples were collected from all cohort members and one stool sample from all bovines to determine outcome measures, which included incidence and intensity of infection for cohort members, and infection rates and intensity of infection for bovines.

### Treatment regime

At baseline all village residents and bovines found positive were treated with praziquantel (PZQ) (humans: 40 mg/kg; bovines: 25 mg/kg) as recommended by WHO[9] until cleared of infection. Previous studies in Poyang and Dongting Lakes showed 85-95% efficacy for a single PZQ dose in humans (40 mg/kg) and water buffaloes (25 mg/kg), with 100% efficacy following re-examination and re-treatment[10-12]. At follow-up all cohort members and all bovines found positive were treated with PZQ (humans: 40 mg/kg; bovines: 25 mg/kg)) as recommended by WHO until cleared of infection[9].

### Snail surveys

A snail survey was performed in April each year to measure the prevalence of infection in snails and the density of infected snails per unit area. This was conducted using the Chinese method of random quadrat sampling (0.11 metres^2 ^sized frames, 20 metres between frames) of the marshland areas.

### Rainfall readings

Rainfall readings were taken every 10 days from two nearby weather stations. These were collected for the duration of the follow-up period (2003-2006).

### Data management and statistical analyses

An MS ACCESS based database was designed specifically for this project and was used for data management[13]. Presence of human infection was defined as at least one egg in all Kato-Katz smears. Egg counts were transformed to eggs per gram and geometric mean intensity was calculated by using the log-transformed egg counts. Confidence intervals (CIs) were calculated using standard formulae based on the binomial distribution (annual incidence of infection) and the lognormal distribution (intensity). Each cohort member was assigned a water contact score for each year preceding infection status assessment. This was determined by adding season-specific sub-scores based on frequency of water contact obtained through the water contact diaries. Formal analyses of annual human incidences, both crude and adjusted (for water contact, using the water contact score), used a generalized linear model (GLM) with a logit link and a binomial error distribution. Generalised equation estimators of parameters with an unstructured variance-covariance matrix were used to account for repeated measures on individuals over time. Analyses used the GENMOD procedure of SAS software (version 9.1; SAS Institute, Inc, Cary, NC) to calculate odds ratios (OR) and 95% confidence intervals (95% CIs).

### Ethical considerations

Written ethical approval for this study was obtained at the national, Sichuan provincial and Shian village, levels; the Human Research Ethics Committee of the Queensland Institute of Medical Research also granted approval for the study. Written informed consent was obtained from all adults and from parents or guardians of minors who were involved in the project. Study participants and bovines identified as positive for schistosomiasis were treated with 40 mg/kg PZQ (25 mg/kg PZQ for bovines)[9].

## Results

### Baseline

#### Human prevalence and intensity of infection

Baseline human prevalence (%) and intensity of infection (geometric mean eggs per gram (GMEPG) in the infected individuals) for *S. japonicum *within Shian village was 42.0% (95% CI: 36.6-47.5) (N = 319) and 32.8 GMEPG (95% CI: 26.9-40.1), respectively (Table 1).

**Table 1:** *S. japonicum *prevalence and intensity of infection in the human cohort from Shian village, Anning River Valley, Sichuan Province, People's Republic of China at baseline in 2002

Human Cohort	Sub-group	N	Prevalence (95% CI)	Intensity EPG (95% CI)
**All**		319	42.0% (36.6, 47.5)	32.8 (26.9, 40.1)

**Sex**	Female	162	41.4% (33.7, 49.0)	34.4 (25.8, 45.8)
	Male	157	42.7% (34.9, 50.5)	31.4 (23.6, 41.7)

**Age**	5-10	56	23.2% (11.8, 34.6)	19.2 (9.1, 40.3)
	11-20	42	23.8% (10.4, 37.2)	32.7 (13.2, 81.0)
	21-30	44	43.2% (27.9, 58.4)	35.4 (21.3, 59.0)
	31-40	79	51.9% (40.6, 63.2)	38.0 (25.2, 57.4)
	41-50	45	57.8% (42.8, 72.8)	28.7 (19.7, 41.8)
	51-60	33	48.5% (30.5, 66.5)	40.0 (22.1, 72.2)
	61-65	19	42.1% (17.7, 66.6)	30.9 (9.3, 102.7)

**Occupation**	Farmer or fisherman	241	47.3% (41.0, 53.7)	35.1 (28.4, 43.3)
	Pre-school	18	22.2% (0.9, 43.5)	16.2 (2.0, 133.5)
	Student	59	27.1% (15.4, 38.8)	24.3 (12.1, 48.8)

#### Human prevalence by sex, age and occupation

There were more females (N = 162) than males (N = 157) in the selected cohort at baseline. Infection prevalence was higher in males (42.7%; 95% CI: 34.9-50.5) than females (41.4%; 95% CI: 33.7-49.0) (Table 1).

The average age of people in the cohort was 32 years. Prevalence within the age groups is shown in Table 1. All age groups had prevalences above 20%, with those between 41-50 years of age having the highest (57.8%; 95% CI: 42.8-72.8).

The majority of the cohort were farmers or fishermen (N = 241), followed by students (N = 59) and pre-school children (N = 18) (Table 1). The highest prevalence was found in the farmer or fisherman group (47.3%; 95% CI: 41.0-53.7) and the lowest was in the pre-school children group (22.2%; 95% CI: 0.9-43.5).

#### Bovine prevalence and intensity of infection

Baseline bovine prevalence (%) for *S. japonicum *was 29.4% (95% CI: 5.3-53.6) (N = 17) and the intensity of infection was low (< 1 GMEPG) (Table 2).

**Table 2:** *S. japonicum *infection rates (95% CI) in bovines at baseline and at follow up

Year	N	Infection Rates (95% CI)
**2002**	17	29.4% (5.3, 53.6)
**2003**	17	11.8% (0.0, 28.9)
**2004**	17	17.6% (0.0, 37.9)
**2005**	16	6.3% (0.0, 19.6)
**2006**	15	0.0% (N/A)

### Follow-up

#### Participant flow

Within Shian village, a cohort of people was selected for follow-up over the course of the study. The flow of these study participants is shown in Table 3. Loss to follow-up per year was small ranging from 0.4% to 10.7%; and the attrition rate for the entire study was 24.8% which is low given the duration of follow-up (4 years). Human treatment coverage was high with 98-100% of those found infected treated with PZQ.

**Table 3:** *S. japonicum *incidence and intensity of infection in the human cohort at follow up

Follow-up	N	Incidence (95% CI)	Intensity EPG (95% CI)
**2003**	285	46.0% (40.1, 51.8)	23.5 (18.4, 30.0)
**2004**	257	31.5% (25.8, 37.2)	20.9 (15.4, 28.4)
**2005**	256	20.3% (15.4, 25.3)	14.2 (10.3, 19.7)
**2006**	240	11.3% (7.2, 15.3)	12.3 (8.0, 18.9)

#### Human incidence and intensity of infection

Over the four years of follow-up, the incidence of *S. japonicum *infection declined from 46.0% (95% CI: 40.1-51.8) in the first year of follow-up to 11.3% (95% CI: 7.2-15.3) in the final year (Table 3). Intensity of infection also decreased from 23.5 GMEPG (95% CI: 18.4-30.0) in the first year of follow-up to 12.3 GMEPG (95% CI: 8.0-18.9) in the final year (Table 3).

**Figure 2:**
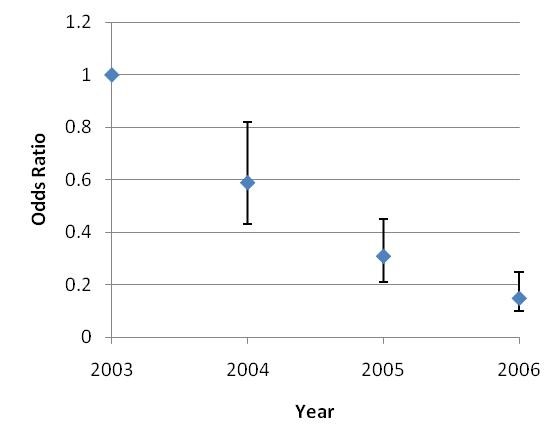
**Annual *Schistosoma japonicum *infection risk in subjects from Shian village (Odds Ratios - adjusted for water contact - with 95% CI)**.

Regression analyses yielding crude and adjusted (for water contact) odds ratios (OR) (Figure 2) for each year of the study were the same. A significant (P < 0.01) downward trend was observed and was in concordance with *S. japonicum *incidence.

#### Human incidence by sex, age and occupation

**Figure 3:**
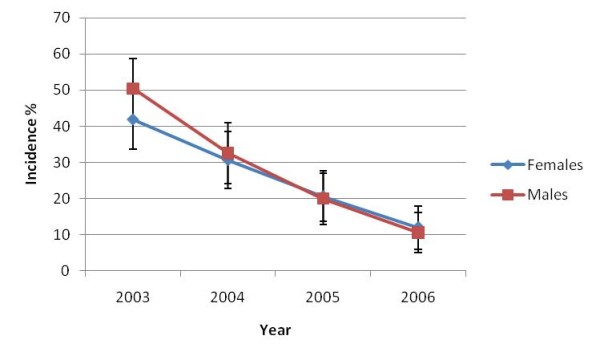
**Human *S. japonicum *incidence over time in males and females (with 95% CI)**.

Over the course of the study, the ratio of cohort males to females remained steady and similar to baseline, although in the final year there were slightly more males (N = 123) than females (N = 117). The *S. japonicum *incidence decreased in females and, especially in males (Figure 3).

**Figure 4:**
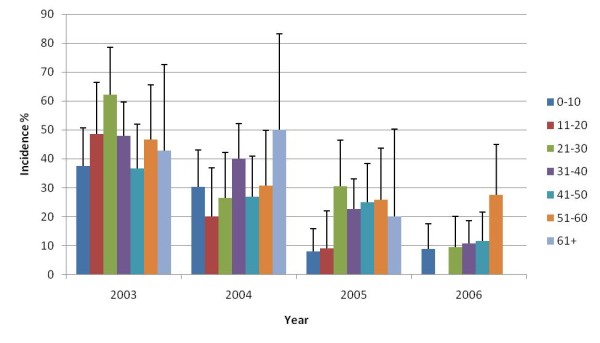
**Human *S. japonicum *incidence over time by age (with 95% CI)**.

**Figure 5:**
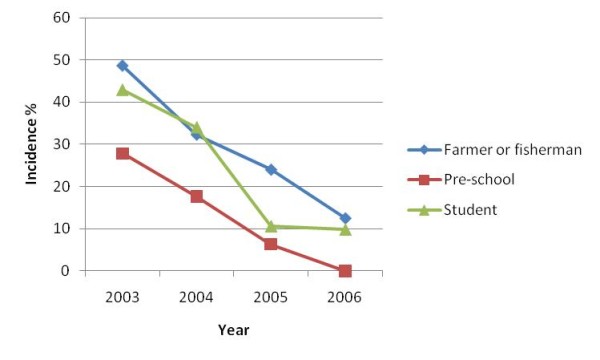
**Human *S. japonicum *incidence over time by occupation**.

In general the incidence decreased from the first to the final year of follow-up in all age groups, although reductions within groups were inconsistent (Figure 4). Similar decreasing trends were evident for occupation during the four years of follow-up, although the incidence plateaued in students in the final year (Figure 5).

#### New human infections and re-infection rates

New infection (those not infected in the previous year) and re-infection (those infected in the previous year) rates were calculated over the duration of the study. In 2003 there was a 40.0% infection rate of new infections, this fell to 8.3% in 2006 (Table 4). Re-infection was greater than new infections over the course of the study although they did follow the same declining trend (53.4% in 2003 to 23.4% in 2006 (Table 4)) as new infections. Both followed the same trend as the overall incidence (Table 3).

**Table 4:** New human *S. japonicum *infection rates and re-infection rates over time

Follow-up	New Infection Rates (95% CI)	Re-infection Rates (95% CI)	P-value
**2003**	40.0% (32.4, 47.6)	53.4% (44.2, 62.7)	< 0.05
**2004**	16.2% (10.1, 22.3)	45.5 (34.1, 56.8)	< 0.001
**2005**	16.1% (10.6, 22.3)	29.6 (19.5, 39.8)	< 0.05
**2006**	8.3% (4.4, 12.2)	23.4 (10.8, 36.0)	< 0.01

#### Bovine infection rates and intensity of infection

Over the four years of follow-up, *S. japonicum *infection rates declined from 11.8% (95% CI: -5.3-28.8) in the first year to zero in the final year of follow-up. In 2004 there was a slight increase in prevalence from 11.8% (2003) to 17.6% (95% CI: -2.6-37.8) (Table 2). The intensity of infection remained low (< 1 GMEPG).

### Dynamics of infected snails

The prevalence and density of infected snails fluctuated substantially over the duration of the study period and consistent trends could not be identified. This was probably because of high levels of snail sampling variability due to spatial aggregation effects that we have observed previously[14].

### Rainfall patterns

**Figure 6:**
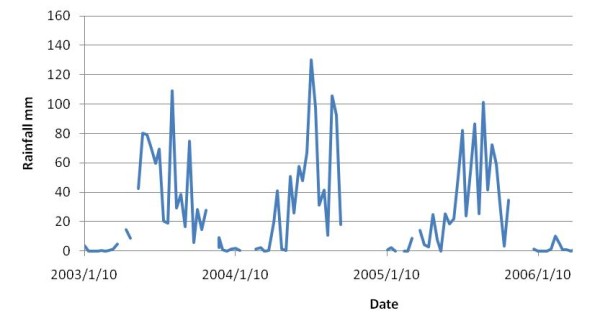
**Rainfall (mm) in Shian village over time**.

Rainfall patterns were similar over the duration of the study with peak rainfall in the summer (Figure 6). There was more rainfall in 2004 and 2006 but there were no reports of major flooding in these two years.

## Discussion and Conclusion

Schistosomiasis transmission in Sichuan (Mode IV) is thought to be quite different to the other transmission modes in the PRC, particularly Modes I - II (Dongting and Poyang lakes) below the TGD[1,2]. Transmission in Sichuan is quite focal and, in the Anning river valley area, the snail habitats lie along the edges of vegetation irrigation ditches, compared with their distribution on the lake shores, on the vast marshlands or along the banks of rivers, canals and water networks in the schistosome-endemic areas below the TGD[15,16]. *Oncomelania hupensis robertsoni*, which harbours *S. japonicum *in Sichuan evolved only at high elevations above the Three Gorges in Sichuan and Yunnan Provinces and is a distinct subspecies to that (*Oncomelania hupensis hupensis*) present below the dam[15]. Furthermore, mixed agriculture is practiced in Sichuan and this does not rely on animal husbandry to the extent operating in the lake and marshland areas. Consequently, bovines, which play a major role in *S. japonicum *transmission below the TGD, may only have a minor role in Sichuan where transmission is thought to be perpetuated through the use of schistosome-egg laden faeces as fertiliser ("night soil")[15-20].

It is predicted that the TGD will change water and sand distributions downstream, hence having a significant impact on ecological systems including the Dongting and Poyang lakes and the canals of Hubei, where *S. japonicum *transmission is generally projected to increase; although it may in fact decrease in some foci[1,3,21]. This is in accordance with other large-scale hydroprojects such as the Gezira-Managil Dam in Sudan, the Aswan Dam in Egypt, the Melkasadi Dam in Ethiopia, and the Danling and Huangshi Dams in China, where schistosomiasis emergences or re-emergences have resulted[22].

The Three Gorges region above the dam is not currently endemic for schistosomiasis due to the previous rapidly flowing waterway that was extremely hostile for snail reproduction and, to date, no oncomelanid snails have been detected there[1,4]. However, the 600 km lake created behind the dam that stretches up-stream now has shorelines suitable in many areas for snail breeding and schistosome transmission. The lake is located between two key transmission zones of *S. japonicum *- Sichuan and Hubei-Hunan. The downstream schistosomiasis-free buffer is only 40 km and the upstream buffer is 500 km. It is anticipated that, in time, these two endemic areas will merge into one, thus potentially spreading the disease into a new location[1,4]. It is projected that the spread of schistosomiasis into this currently non-endemic area would be expedited by infected humans travelling on the newly created waterways and by the introduction of oncomelanid snails[1,4]. Once schistosomes are established in the lake they would be difficult to eliminate[23].

We hypothesised that the TGD would have little or no impact on transmission in areas currently endemic for *S. japonicum *in Sichuan Province; and that the creation of new endemic areas would take upward of 10 years as the water flow slows down and silt deposits settle, forming new marshland areas. Snail dispersal and population movements will also be required to introduce schistosomes into these areas.

We undertook longitudinal surveillance from 2002 to 2006 in Shian village in the Anning river valley of Sichuan Province, to determine the immediate impact upstream of the construction of the TGD on schistosome transmission. The results showed that the incidence of *S. japonicum *infection in the selected human cohort decreased by three quarters from 46% in 2003 to 11.3% in 2006 (Table 3). A significant (P < 0.01) downward trend was also evident in the yearly adjusted (for water contact) odds ratios (Figure 2).

The decrease in human incidence observed in Shian village may be attributed to the annual PZQ treatment administered to infected individuals (ethical reasons). Torrential rains that sometimes occur in regions of high terrain inclination (Sichuan) can wash snails down to lowland sites thus decreasing transmission[20], but this had not occurred in Shian (Figure 6). However, the concurrent decline in bovine infection rates observed in Shian suggests that this may also have contributed to the decline in human incidence. Studies have shown that bovines are the major transmission source of human schistosomiasis in the Dongting and Poyang lakes and marshland areas and that interventions targeting bovines reduce the incidence of human infection[17,18]. This study of Shian village suggests that bovines may also be important for human transmission in Sichuan, which is contrary to previous reports that downplay the role of animals, particularly bovines, in schistosome transmission in Sichuan[15,16]. It is noteworthy as well that a recent article by Zou *et a*l, shows that domestic livestock, particularly bovines, are important in schistosomiasis transmission in Yunnan Province[24]. The bovine involvement in transmission may be through either direct contamination of the environment or through the use of their schistosome-egg laden faeces for crop fertilisation. Nevertheless, they should be targeted by control programs in this region.

The substantial decrease in human (75%) and bovine (100%) incidence observed in Shian village can probably be attributed to the annual human and bovine PZQ treatment of positives; as seen in drug (PZQ) intervention studies in other parts of PRC[18]. It should be noted no decrease in exposure was seen as that water contact patterns did not change over the duration of the study, indicated by the similarity in crude and adjusted (for water contact) OR. If an increase in schistosome transmission had occurred as a result of the TGD, it would be of negligible size compared to the treatment induced decline seen here. It appears therefore that the construction of the TGD had virtually no impact on schistosomiasis transmission in Shian village over the period of study.

Another finding of note is that despite the trend of decline, in accordance with overall incidence, re-infection rates were higher than new infection rates over the duration of the study (Table 4), thus suggesting that many of the same people were becoming infected in this village. This gives insight for future control, as interventions, particularly chemotherapy and health education, can be directed towards this group of people.

A recent article by Liang et al [25] demonstrated that schistosomiasis is re-emerging in Sichuan Province, particularly in previously endemic areas, classified recently by the Chinese authorities as under transmission control (*S. japonicum *prevalence in humans and bovines is <1%) or transmission interruption (*S. japonicum *prevalence in humans and bovines is <0.2%). It is likely that this re-emergence, signalled by the occurrence of acute infections, is due to changes in the socio-political and economic (diminished funding and awareness for schistosomiasis control) climates and in the local environment and surveillance systems and increased human mobility from non-endemic areas and inappropriate surveillance[25]. These changes have major implications for future control efforts especially with the future threat of the development of new schistosome-endemic areas due to construction and completion of the TGD[1,3,4].

PZQ treatment alone will not be able to mitigate schistosome re-emergence, prevent increases in transmission or avert the creation of new endemic areas. This is due to the inability of the drug to prevent re-infection[26]. This is exemplified by the current study where, even after 5 years of annual PZQ treatment, despite a decrease in human incidence, it was still over 10% (Table 3). As mathematical modelling has shown, incidence will rebound to pre-treatment levels on its cessation[27]. Therefore, a multi-component, integrated control program[1,26] will be required to combat the spread of schistosomiasis and achieve the Chinese Government's goal of reducing the prevalence of *S. japonicum *to less than 1% by 2014[28]. Indeed, pilot programs are already in place in the PRC where new integrated strategies have been instigated to prevent schistosome eggs contaminating the environment. These emphasise health education, access to clean water and adequate sanitation, mechanisation of agriculture, fencing of water buffaloes, snail control, chemotherapy, and even future vaccination of livestock [28-30].

## Competing interests

The authors declare that they have no competing interests.

## Authors' contributions

DPM, YSL, GMW, ZF and JGG conceived the study; RZ, YZ, DCQ, HQZ, WW, YSL, GL, ZF, DPM, GMW, JGG and DJG carried out research planning, stool examinations and questionnaire work; DJG, GMW, APT, RZ, RSL and DPM analysed the data; and ZR, DJG, APT and DPM wrote the manuscript with input from all other authors.
